# Pangenome analysis of Enterobacteria reveals richness of secondary metabolite gene clusters and their associated gene sets

**DOI:** 10.1016/j.synbio.2022.04.011

**Published:** 2022-05-06

**Authors:** Omkar S. Mohite, Colton J. Lloyd, Jonathan M. Monk, Tilmann Weber, Bernhard O. Palsson

**Affiliations:** a The Novo Nordisk Foundation Center for Biosustainability, Technical University of Denmark, Kongens Lyngby, 2800, Denmark; b Department of Bioengineering, University of California San Diego, La Jolla, CA, 92093, USA

**Keywords:** Pangenome analysis, Workflow, Secondary metabolites, Colibactin, Enterobacteria, Secretion systems, BGC, Biosynthetic gene cluster, GCF, Gene cluster family, PKS, Polyketide synthase, NRPS, Non-ribosomal peptide synthetase, RiPP, Ribosomally synthesized and post-translationally modified peptide, T6SS, TypeVI Secretion System, T4SS, Type IV Secretion System

## Abstract

In silico genome mining provides easy access to secondary metabolite biosynthetic gene clusters (BGCs) encoding the biosynthesis of many bioactive compounds, which are the basis for many important drugs used in human medicine. However, the association between BGCs and other functions encoded in the genomes of producers have remained elusive. Here, we present a systems biology workflow that integrates genome mining with a detailed pangenome analysis for detecting genes associated with a particular BGC. We analyzed 3,889 enterobacterial genomes and found 13,266 BGCs, represented by 252 distinct BGC families and 347 additional singletons. A pangenome analysis revealed 88 genes putatively associated with a specific BGC coding for the colon cancer-related colibactin that code for diverse metabolic and regulatory functions*.* The presented workflow opens up the possibility to discover novel secondary metabolites, better understand their physiological roles, and provides a guide to identify and analyze BGC associated gene sets.

## 1 Introduction

Secondary metabolites produced by a range of microorganisms display medicinally and industrially important properties, and mediate microbe-host and microbe-microbe interactions. Secondary metabolite biosynthesis often involves mega-enzymes such as polyketide synthases (PKS) and non-ribosomal peptide synthetases (NRPS) that are encoded by large biosynthetic gene clusters (BGCs). Recent advances in genome sequencing technology and genome mining tools revealed an unexplored richness and diversity of BGCs encoding secondary metabolites [[1], [2], [3], [4]]. However, understanding the interaction between biosynthesis of secondary metabolites and other functions encoded in the genome outside the cluster region remains a challenge. Recently, the availability of a large number of genomes from the same species allowed for pangenome analysis revealing intra-species diversity, such as metabolic capabilities [5,6]. Such pangenome analysis involving the comparison of a group of genomes carrying a particular BGC against others might help us bridge this gap between secondary metabolism and other functions.

The focus of many genome mining based studies has been well-established secondary metabolite producers, such as bacilli, actinobacteria, or myxobacteria [7,8]. Compared to many of these popular secondary metabolite producers, *Escherichia coli* or other enterobacteria have a larger availability of sequenced genomes, higher quality of genome annotations, comprehensive curated databases, and extensive tools for data analysis. Enterobacteria are known to produce secondary metabolites that include metal ion chelators like enterobactin, yersiniabactin [9,10], the colon cancer-related genotoxin colibactin [[11], [12], [13]], the antibiotic althiomycin [14], the red pigment prodigiosin, and the biosurfactant serrawettin W1 [15]. In particular, a PKS-NRPS type BGC from certain *E. coli* strains and other enterobacteria is responsible for the biosynthesis of colibactin associated with colon cancer [12,13]. Some enterobacteria associated with nematodes like *Photorhabdus sp.* and *Xenorhabdus sp.* are known to produce a diverse range of secondary metabolites including proteasome inhibitor luminmycin, antibiotic xenocoumacin, anthraquinone pigments, and others [[16], [17], [18], [19]]. More recently, darobactin was isolated in a *Photorhabdus* isolate that selectively kills gram-negative pathogens [20]. Because of these important secondary metabolites, the availability of a large number of high-quality genomes, and lack of large genome mining studies of enterobacteria compared to other soil bacteria, we selected this group of bacteria for the analysis presented here.

**Fig. 1:**
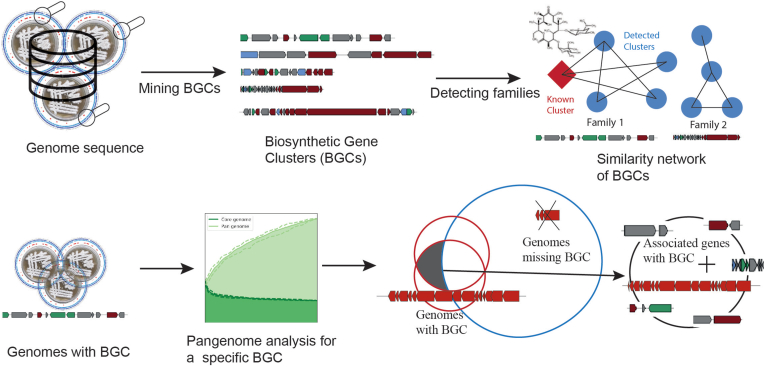
Schematic representation of the presented large-scale workflow, integrating genome mining and pangenome analysis tools A large set of genomes were mined for BGCs encoding for secondary metabolite biosynthesis using antiSMASH [3]. The diversity of detected clusters and the identification of known BGCs were examined using BiG-SCAPE [4] and the MIBIG database [26]. Further, a pangenome analysis was carried out for a set of genomes having the same BGC. Common genes across genomes possessing a particular BGC are then compared against those missing that BGC to identify a set of genes that might have a functional association with the presence of a BGC.

Here, we aim to combine strengths of genome mining and pangenome analysis to assess the potential of enterobacteria to produce diverse secondary metabolites and understand the association between secondary metabolism and other biological functions (Fig. 1). In this integrated workflow, we analyzed a large set of high-quality complete genomes of enterobacteria using the software antiSMASH to investigate their secondary metabolite biosynthetic potential [3]. Further, distinct families of BGCs were identified based on a sequence similarity network generated using BiG-SCAPE [4]. Next, we selected 60 *Escherichia* genomes possessing a clinically important colibactin cluster and performed core- and pangenome analyses. The pangenome analysis identified a colibactin associated set of genes that might have associations with the function of colibactin clusters (e.g., a putative secretion system) that could reveal cryptic mechanisms of colibactin activity.

## 2 Materials and methods

### 2.1 Collection and quality assessment of enterobacterial genomes

We collected 4,035 genome entries from the PATRIC database v3.6 [21] of Enterobacteriales which are annotated as ‘complete’ as of 15 June 2020. The quality of genome assembly is important for better prediction of genome mining as well as for the pangenome analysis. Recently, studies have shown the selection of a high quality and appropriate starting dataset is of importance in pangenome studies [22,23]. In our study, we curated our dataset following several criteria for the genome mining part and further focused on only 60 selected genomes with BGC of interest for the pangenome analysis. First, a total of 47 genomes with multiple contigs in the chromosome were removed from the analysis. We found that 59 strains had more than one genome in the dataset. In order to remove the redundancy, we selected only one genome for each of these organisms resulting in 3896 genomes in the curated dataset (Dataset S1). To further curate the data, we manually removed 7 genome entries due to irregular genome size (Dataset S1). Finally, a genome with a PATRIC identifier ‘562.58112’ was manually removed since the sequence data was not available at the PATRIC database. The distribution of genome size against GC content showed that there are very few genomes that are outliers when compared to the average genome size of a particular genus (Fig. S1). Genomes from other genera including endosymbiont genera such as *Buchnera*, which have relatively lower genome sizes, are not removed from the analysis. Thus, the final dataset contained 3,889 genomes spread across 57 different genera with the majority belonging to *Escherichia*, *Salmonella,* and *Klebsiella* (Dataset S1).

### 2.2 Genome mining of secondary metabolites BGCs across enterobacteria

antiSMASH v5 was used for genome mining of secondary metabolite BGCs in 3,889 enterobacterial genomes [3]. We used ‘.gff’ files downloaded from the PATRIC database for gene annotations with the antiSMASH run. The final data set contains 13,266 BGCs detected across genomes of enterobacteria (Dataset S2). For genus-specific analysis and visualization, we considered the top 15 genera with the highest total number of clusters per genus. The remaining 36 genera are all classified as ‘Other’. Similarly, we considered the top 15 most occurring types of the BGCs as defined in antiSMASH output and classify the remaining 72 BGC types as ‘Other’ (Dataset S2).

### 2.3 Phylogenetic tree of selected enterobacterial strains with different BGC distributions

**Fig. 2:**
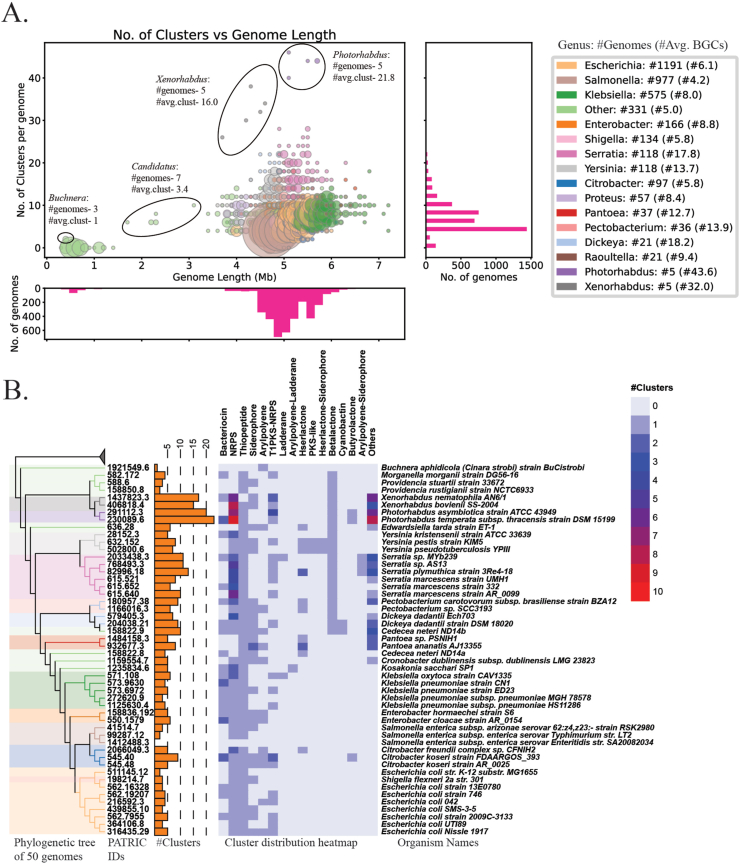
Distribution of BGCs detected across diverse genera of enterobacteria A. A scatter plot showing the association between the numbers of BGCs detected across 3889 enterobacterial genomes versus genome length (Fig. S1, Dataset S1 and S2). The size of the circles represents the number of genomes, colors denote major genera. Associated histograms represent the distribution of BGCs in genomes (right) and lengths of genomes (bottom). The number of genomes per genera and average number of BGCs are denoted along with the color legend. B. Distribution of presence of BGCs of various types across 50 selected genomes from 21 diverse genera of enterobacteria. The phylogenetic tree was generated based on all shared proteins and using maximum likelihood algorithm (RAxML) with an out-group of 5 genomes from neighboring clades of Gammaproteobacteria (Dataset S2). The leaves of the tree are colored based on different genera of enterobacteria. Bar charts with the number of BGCs per genome, PATRIC genome accessions, and organism names are also displayed (Dataset S2).

A set of 50 genomes was selected from various genera with different distributions of BGC types as detected through genome mining (Dataset S2). A phylogenetic tree was constructed with PATRIC [21] services for tree building based on all shared proteins and using maximum likelihood algorithm (RAxML) [24]. The out-group set used for the tree construction involved five genomes of neighboring clades in Gammaproteobacteria from the PATRIC database, which are 1) *Mannheimia succiniciproducens* MBEL55E; 2) *Pasteurella multocida* subsp. multocida str. Pm70; 3) *Photobacterium profundum* SS9; 4) *Vibrio fischeri* ES114; and 5) *Vibrio cholerae* O1 biovar El Tor str. N16961. The phylogenetic tree was visualized using iTOL v5 (Interactive Tree of Life) [25], with additional panels representing a bar chart of total BGC distribution and heatmap of BGC type distribution across all genomes (Fig. 2).

### 2.4 Sequence-based similarity network of BGCs detected in Enterobacteria

BiG-SCAPE [4] was used to generate a sequence-based similarity network of 13,266 detected clusters. Here, ‘*--mibig’* parameter was used for comparison against 1795 known BGCs from the MIBIG database [26]. For the similarity index between any two clusters, we used *raw_index* metric from BiG-SCAPE output. We tried 3 different cutoffs of 0.3, 0.5, 0.7 on *raw_index* to define the similarity network. As the sequences of many BGCs are very similar to each other, we used a cutoff of 0.3 for the final network (Dataset S3). The comparison was carried out separately for the major classes of BGCs by using the parameter *--hybrids-off*.

**Fig. 3:**
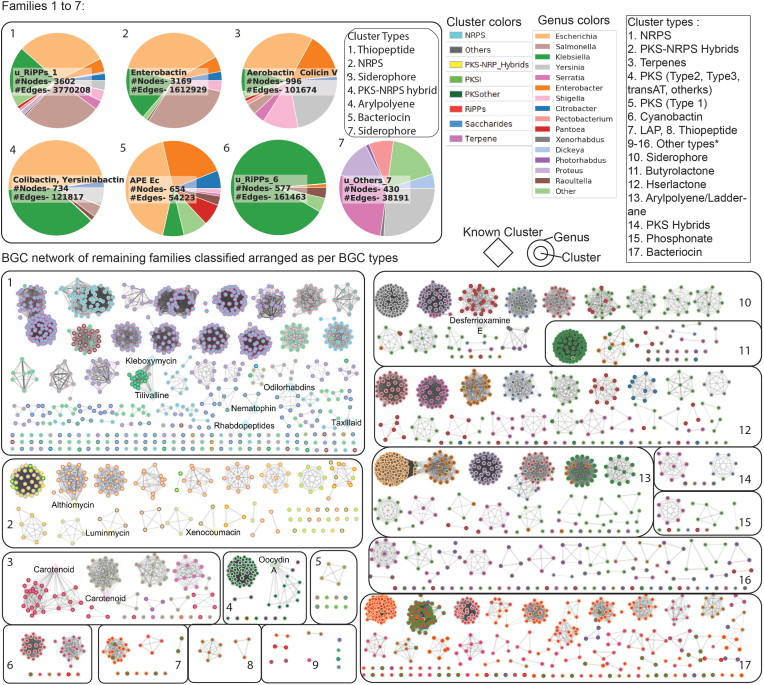
Global network view of BGCs across Enterobacteria Sequence similarity network of 13266 BGCs with 1795 known BGCs from the MIBIG database reveals 252 distinct families or disconnected components of the network (Dataset S3). The seven largest families with highly connected networks are represented by pie charts. The number of nodes, number of edges, and any known MIBIG BGCs are shown in the pie chart. Different colors denote different genera. For the remaining smaller families, we used Cytoscape to visualize the networks which are grouped according to the class of BGCs in 1–17 as described in the legend. Each node represents a biosynthetic gene cluster, where the color of the inner circle denotes the class of the BGC and the color of the outer circle denotes different genera. Edges between two BGCs denote the dissimilarity metric detected using the BiG-SCAPE pipeline. Known BGCs from MIBIG are denoted by diamond-shaped nodes. For distribution of families across genomes, see Fig. S2.

The distinct connected components of the similarity network, based on a cutoff of 0.3 on *raw_index*, are denoted as distinct families of BGCs. Using the similarity network, 599 completely distinct families were detected with 347 of them being unique families as represented by singleton nodes in the network. The distribution of BGC families across genomes of different genera is represented by a heatmap (Fig. S2). As some of the BGC families are more frequent than others in enterobacteria, the distribution of BGCs in the top 7 largest families are visualized using pie-charts (Fig. 3). The networks for remaining smaller families are visualized using Cytoscape [27] and are categorized based on BGC classes (Fig. 3). Further, we analyzed some of the families (family 2 and family 3) by calculating the adjacency matrix of the network to understand the intra-family diversity in BGC contents (Fig. S3).

### 2.5 Variations within colibactin gene cluster family

**Fig. 4:**
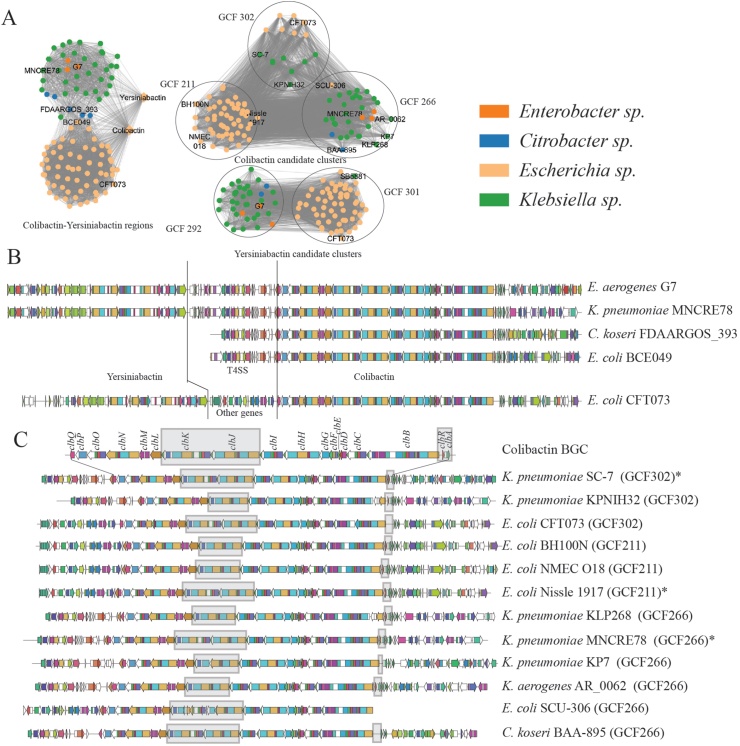
Colibactin containing BGC family and genetic variation across genera A. Similarity network of candidate clusters from the colibactin BGCs from across multiple genera (denoted by colors). B. Genetic structure of colibactin BGCs across different genera. For *Citrobacter koseri*, the yersiniabactin and colibactin gene clusters occur at different positions in the genomes. The different intermediate regions between the colibactin and yersiniabactin are shown. C. Variations within colibactin biosynthetic genes (*clbA-clbS*) across selected BGCs (find a detailed comparison in Fig. S4).

The total of 105 BGCs similar to known colibactin and yersiniabactin clusters were further split into 303 candidate clusters using antiSMASH v5 annotation. A similarity network of these 303 candidate clusters was calculated using BiG-SCAPE to separate the colibactin BGC from yersiniabactin BGC (Fig. 4, Dataset S3). The genetic structures of the entire colibactin and yersiniabactin coding regions from different genera were further compared to investigate inter genera diversity of BGCs (Fig. 4). Further, selected candidate clusters coding for colibactin alone were compared to find the variation within colibactin biosynthetic genes (Fig. 4). Further, bidirectional best blast hits were calculated for all biosynthetic genes (*clbA* - *clbS*) across 105 colibactin BGCs (Fig. S4). Different alleles of biosynthetic genes and their distribution across 105 BGCs were calculated based on amino acid sequences. Lastly, the amino acid sequences for different alleles of some of the biosynthetic genes were aligned (Fig. S4).

### 2.6 Characterization of colibactin associated gene sets for *Escherichia*

**Fig. 5:**
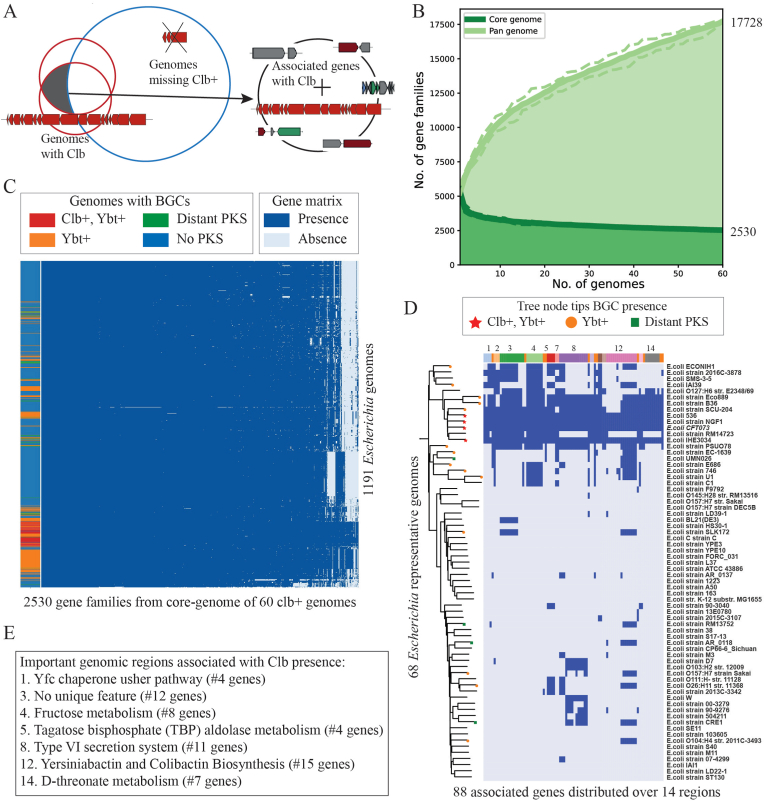
Pangenome reconstruction of 60 colibactin containing *Escherichia* genomes and detection of associated genes with colibactin BGC A. Schema describing comparison of core-genome of colibactin containing genomes against those missing colibactin to detect a set of genes associated with the presence of colibactin. B. 60 genomes possessing colibactin BGC are selected for the reconstruction of the core-genome (common across all genomes) and pan-genome (total genes). C. Gene presence/absence heatmap of 2530 core genes (columns) as compared against all 1191 genomes of *Escherichia* in the phylogenetic order (columns) (Dataset S4). Columns represent genomes divided into four groups: i) both colibactin and yersiniabactin clusters (60); ii) only yersiniabactin cluster (176); iii) PKS-NRPS clusters that are distantly similar to yersiniabactin or colibactin (24); and iv) none of the PKS clusters (546). Here, the gene presence is defined if the homolog identity was higher than 80%. D. Phylogenetic tree of 68 selected genomes of *Escherichia* based on ANI calculation and distribution of 88 associated genes. The columns are grouped by colors depending on the presence of genes in different regions/operons in the genome. The few selected regions are further described (Dataset 4). E. List of important genomic regions from an associated set of genes with common biological functions.

We selected 60 genomes of *Escherichia sp.* possessing the colibactin gene cluster for pangenome analysis. The pangenome reconstruction was carried out using Roary software [28] (Fig. 5, Data S4). For further investigation, we selected genes from the core genome that are commonly present in the 60 genomes with a colibactin BGC. To analyze the specific functions associated with the colibactin BGC, we compared the 2,530 genes coding for proteins from the core genome against all of the 1,191 genomes using bi-direction best hits using diamond (Data S4). A gene presence-absence heatmap was generated with genes with greater than 80% identity defined to be similar (Fig. 5). The set of 88 genes that are present across all of colibactin containing genomes and missing in more than 90% of no pks containing genomes were further investigated for their putative role in the function of colibactin BGC (Fig. 5, Dataset S4). The set of associated genes that appear next to each other in the genome is defined as a genomic region. In total, 14 such genomic regions were defined incorporating 77 of the 88 associated genes. Many of the regions with genes encoding for a pathway or cluster of genes having similar functional roles are manually assigned a common functional category (Fig. 5). For example, the region number 14, including some of the genes involved in d-threonate related genes, was assigned as a d-threonate metabolism region. As part of the workflow, the sequence conservation of genes in selected regions is visualized using a heatmap of blastp percentage identity values (PID) (Fig. S5). A heatmap of PID values of these homologs across all *Escherichia* genomes in phylogenetic order was calculated to see the conservation of sequences across colibactin containing genomes.

## 3 Results

### 3.1 Large scale genome mining and pangenome analysis workflow

Here, we present a large-scale systems biology workflow integrating various genome mining and pangenome analysis tools to analyze a large number of genomes to find diversity and variations in BGCs and associated genes.

In the first stage of comparative BGC analysis, we selected a manually curated dataset of only complete genomes from the PATRIC database [29] for genome mining. The downloaded genomes were then analyzed using the genome mining software antiSMASH [3] to detect secondary metabolite BGCs. The distribution of BGCs of various types spread across several genera was investigated in detail. In the next step, the set of detected BGCs and the BGCs with associated known products from the MIBIG database [30] were analyzed together using BiG-SCAPE [4]. The generated similarity networks were then analyzed using the network visualization tool Cytoscape to investigate the number of different biosynthetic gene cluster families (GCFs) present across the dataset. The presence of BGCs with associated known products from the MIBiG database was used to understand the GCFs with predicted known vs. unknown products. Here, we note that the estimation of the number of known GCFs is limited by the described associated products in the MIBIG catalog of BGCs. The variations within selected abundant GCFs were visualized and analyzed in greater detail using BGC alignments, heatmaps representing adjacency matrices of GCF networks, and heatmaps representing presence or absence of core genes of BGCs across the GCF. Further, amino acid variations within some of the key genes were also analyzed, providing a detailed workflow to carry out comparative analyses at various stages. This part of this workflow was also applied to investigate phylogenetic distribution of BGCs across Bacillus subtilis species complex in our earlier study [31].

In the second stage of pangenome analysis, we selected a group of genomes from a genera that contained a BGC of interest. We carried out core and pangenome analysis on this set of genomes with BGC-present using Roary [28]. In the next step, we compared all the genes in the core genome of BGC-present strains against all genes from all the genomes in that genera. Thus, the constructed gene presence-absence matrix was further investigated to detect conserved sets of genes within BGC-present genomes against BGC-absent genomes. The distribution of associatively present genes were also analyzed against the phylogenetic tree to understand evolutionary relationships. Thus the comprehensive workflow presented here could guide us towards finding possible associations of BGCs with the rest of the genes spread across the genomes and how they might have evolved together.

### 3.2 Characterization of secondary metabolite gene clusters across closely related enterobacteria

In this study, we applied the presented integrated workflow on the selected group of enterobacterial genomes to investigate distribution of BGC types. First, we set out to examine 3,889 complete genomes from 57 different genera of enterobacteria downloaded from the PATRIC database [21] in order to investigate the number and kinds of secondary metabolic BGCs the different genera might contain (Dataset S1). Using antiSMASH [3], we detected 13,266 BGCs across 3,889 genomes with the global average of 3.41 BGCs per genome across enterobacteria (Dataset S2). It should be noted that the dataset of genomes is unevenly distributed over 57 genera and is represented primarily by the genomes from genera *Escherichia* (1,191), *Salmonella* (977), *Klebsiella* (575), *Enterobacter* (166), *Shigella* (134), *Yersinia* (118), and *Serratia* (118) (Dataset S1). The average number of clusters varies across different genera of enterobacteria (Fig. 2, Dataset S2). We observed that *Photorhabdus*, *Xenorhabdus,* and *Serratia* are some of the genera with a relatively higher richness in BGCs and have also been previously described to produce diverse secondary metabolites [14,15,17,18,32]. There is a genus-dependent association between the number of BGCs and the size of the genome (Fig. 2). Interestingly, three of the very small genomes (length <0.5 Mb) from *Buchnera*, endosymbionts of aphids, also carry a BGC from the aryl polyene class, which are polyketide-derived pigments (Fig. 2).

The detected BGCs are of 87 different types, inferred through the rule-based antiSMASH BGC detection logic, which includes 31 individual and 56 hybrid BGC types (Dataset S2). Various BGC types include those encoding for NRPS (4,026), thiopeptides (3,618), bacteriocins (1,062), siderophores (1,013), PKS-NRPS hybrids (897), arylpolyenes (721), and 58 other types of BGCs (1,929) spread throughout diverse genera (Dataset S2). A phylogenetic tree of 50 selected genomes from different genera (Dataset S2), constructed using all shared proteins and the maximum likelihood algorithm (RAxML) [24], displayed an evolutionary relation of BGCs across enterobacteria (Fig. 2). Most genomes possessed at least one NRPS BGC encoding siderophore (enterobactin or a similar derivative) and one uncharacterized thiopeptide BGC. The antiSMASH annotated thiopeptide BGC did not contain all essential parts of a typical thiopeptide, but contained a gene encoding the YcaO superfamily of proteins involved in post-translational modifications of ribosomal protein S12 in *E. coli* [33]. Enterobacterial genomes are observed to have higher diversity in NRPS type BGCs especially in *Photorhabdus* (avg. 7.8 BGCs), *Xenorhabdus* (avg. 7.2 BGCs), and *Serratia* (avg. 4.1 BGCs).

We also noticed that many other cluster types are distributed differently across different genera of enterobacteria (Fig. 2). The *Salmonella* genus has the least diversity compared to other enterobacteria. Among 1,191 Escherichia genomes, 310 genomes contain four BGCs, 121 genomes contain five, and 18 genomes contain six BGCs. These BGCs in *Escherichia,* apart from NRPS and YcaO containing RiPP, belong to various types including bacteriocins, siderophores, aryl polyenes, aryl polyene-ladderane hybrids, and type 1 PKS-NRPS hybrids. Bacteriocins are most commonly observed in *Klebsiella* (Fig. 2). The most common bacteriocin in *Klebsiella* BGC contains the *dsbA* gene with a domain similar to the circular bacteriocin family (TIGR03651), however the BGC appears to be incomplete. *Yersinia* and *Serratia* genera have much higher diversity than other abundant genera in our dataset, with average clusters of 6.8 and 8.9, respectively. Specifically, we observed that non-NRPS derived siderophores, homoserine-lactones, and PKS-like BGCs are highly common across *Yersinia*, whereas *Serratia* genomes have multiple NRPS clusters. Most of these BGCs detected in enterobacteria encode for secondary metabolites that play an important role in microbe-microbe and microbe-host interactions. Thus, with the use of genome sequence data and genome mining tools, we highlight that the enterobacteria contain a greater richness of BGCs than previously realized.

### 3.3 Sequence based similarity network reveals at least 584 uncharacterized families of BGCs in Enterobacteria

Next, we asked how many of the detected BGCs are distinct and how many of them have been assigned to well-characterized secondary metabolites. We used the BiG-SCAPE pipeline [4] to compare sequences of 13,266 detected BGCs and 1,795 known BGCs from the MIBIG database [26]. BiG-SCAPE compares protein domain sequences of BGCs to define a weighted combination of domain sequence similarity, Jaccard index, and adjacency index as a raw distance metric to generate a sequence similarity network (default cutoff of 0.3 was used for network, see Methods). The generated sequence similarity network constituted 252 distinct connected subnetworks (here called gene cluster families (GCFs)) of BGCs among enterobacterial genomes (Fig. 3), with 347 BGCs being singletons (Dataset S3). Surprisingly, only 15 GCFs were associated with 24 previously characterized BGCs from the MIBIG database, whereas the remaining 237 GCFs and 347 singleton BGCs might encode for novel secondary metabolites.

The seven most common GCFs were distributed among diverse genera, whereas the remaining 245 GCFs represented by a network were mostly genus-specific (Fig. 3). Some of the most common GCFs code for known compounds like enterobactin, aerobactin, colicin V, colibactin, yersiniabactin, colibactin, and APE Ec (*E. coli* aryl polyene). The GCF presence/absence heatmap further displays how GCFs are distributed across genomes from various genera (Fig. S2). We found that the *Serratia* genomes alone contained BGCs spread across 62 GCFs. Some of the other genera with a high diversity of GCFs included *Dickeya* (23 GCFs), *Photorhabdus* (23 GCFs), *Pantoea* (20 GCFs), *Pectobacterium* (18 GCFs), and *Xenorhabdus* (16 GCFs), despite a smaller number of genomes in the dataset. Thus, the comparative analysis of BGCs across species can lead us to novel insights about activity of secondary metabolites.

### 3.4 Intra-family comparison reveals the variations within BGC families

Within the workflow, it is possible to focus on individual BGC families and obtain in-depth information. It is known that different enterobacteria produce a variety of siderophores derived from enterobactin, such as salmochelin, that act as a bacterial evasion mechanism against the mammalian protein siderocalin in *Salmonella* strains and some uropathogenic *E. coli* [34]. Additionally, intra-family genetic diversity among aerobactin and salmochelin siderophores was shown to be associated with different mobile genetic elements [35]. Therefore, we analyzed genetic variations of particular families through adjacency matrices to get an overall view of finer clustering of BGCs. Adjacency matrices calculated for some of the large families (family 1 and 2) reveal intra-family diversity among BGCs (Fig. S3). The clustering algorithm of BiG-SCAPE further detected several GCFs within these two families. We selected one BGC per GCF defined by this clustering algorithm and reran BiG-SCAPE on these selected BGCs to get an overview of intra-family variations (Fig. S3). The YcaO gene appears to be conserved across the largest RiPP family. This BGC appears to be highly conserved with minor changes in genes that are likely not part of biosynthesis. The biosynthetic genes of the second largest family of enterobactin also appear to be widely conserved, apart from the gene *fepE* that was missing in part of the BGCs. The gene *fepE* is involved in ferric-enterobactin cytoplasmic membrane transport in conjunction with other genes *fepC* and *fepD* [36].

In another example, we examined the genetic structural variations in the fourth-largest family of PKS-NRPS BGCs that had 732 clusters. Among them, 105 and 328 BGCs were directly similar to colibactin and yersiniabactin respectively, and 74 of them shared similarity with both colibactin and yersiniabactin. The remaining 375 BGCs were distantly similar to colibactin or yersiniabactin (not immediate neighbors in the network). We specifically selected 105 BGCs that are directly similar to the colibactin MIBIG entry (MIBiG ID: BGC0000972) for further comparison. These BGCs are present across diverse genera like *Escherichia*, *Klebsiella*, *Enterobacter*, and *Citrobacter*. Recent studies have shown a tight correlation between colibactin and yersiniabactin evolution and expression [[37], [38], [39], [40]]. The yersiniabactin BGC is observed to be present whenever colibactin is present across genera as also seen earlier [41], with exception of *E. coli* BCE049 and *K. pneumoniae* 11492 that only contained colibactin BGC. Additionally, yersiniabactin and colibactin clusters are located close to each other in most cases, with the exception of three of *Citrobacter koseri* strains where they were located apart in the genome (Fig. 4). This also means that in most cases, antiSMASH will detect both clusters as one larger region. Hence, we used the candidate cluster feature of antiSMASH v5 to separate the two clusters. We extracted 303 candidate clusters from 105 BGC regions encoding colibactin. The region located between colibactin and yersiniabactin clusters varied across genera (Fig. 4). The intermediate region between the two clusters suggested the presence of genes coding a type IV secretion system (T4SS) in *Klebsiella sp.* and *Enterobacter sp,* as was also observed previously [39]*.* In *Citrobacter sp.*, where yersiniabactin and colibactin BGCs are in separate locations, T4SS encoding genes were still observed next to the colibactin core biosynthetic genes. Interestingly, no such genes related to secretion systems were observed in the cluster region for *Escherichia sp*.

As part of the workflow, we carried out a phylogenetic analysis of 104 genomes containing colibactin BGC using autoMLST (Fig. S4) and calculated bidirectional best blastp hits of colibactin biosynthetic genes across 104 genomes (Dataset S3). We found that as many as 18 genomes were missing the gene *clbR,* coding for a key transcriptional activator of colibactin gene expression [42]. Additionally, 11 of these 18 genomes also lacked the gene *clbA,* coding for a phosphopantetheinyl transferase, putatively activating the colibactin PKS-NRPS [38]. To investigate further variations in other genes, we calculated different alleles for each of the biosynthetic genes across the genomes (Dataset S3). We found that for most of the genes, one single allele was conserved across most strains, whereas two or more alleles were conserved in different genomes for genes such as *clbS, clbQ, clbK, clbJ,* and *clbH* (Fig. S4). In order to investigate variation in different alleles, we further aligned the amino acid sequences for these different alleles. For example, the amino acid sequence variation between four alleles of *clbS* include K to R at position 12 (*clbS_allele2* and *clbS_allele3*), L to I at position 37 (*clbS_allele3*), and D to Y at position 129 (*clbS_allele4*) (Fig. S4). In general, the variations appear to be minimal in most cases. Our detailed comparative analysis shows the natural variations in the colibactin genes and provides a platform for future experimental studies to verify the effects of genetic variations.

### 3.5 Pangenome characterization of *Escherichia* genomes reveals a colibactin associated set of genes

We next asked if the comparison of as many as 1,191 genomes of *Escherichia sp.* from the dataset could guide us to detect specific genetic and functional associations with the presence of a particular BGC. Here, we investigated a total of 60 *Escherichia* genomes possessing colibactin using pangenome reconstruction and genome-wide association techniques (Dataset S4) [5,43,44]. For 60 colibactin-containing *Escherichia* genomes, a total of 17,728 different genes were detected in the pan-genome (total genes found across genomes), which was reconstructed using Roary [28] (Fig. 5, Data S4). The core-genome (genes shared among genomes) was composed of 2,530 genes. Conversely, 5,804 genes were uniquely present in only one of the genomes. Next, we compared sequences of 2,530 genes from the reconstructed core-genome against all 1,191 *Escherichia* genomes present in the dataset using bidirectional best blastp hits (Fig. 5, Data S4). The gene presence/absence heatmap (Fig. 5) revealed a set of 88 genes that are present in all 60 colibactin-containing genomes and are absent in more than 90% of the genomes missing both colibactin and yersiniabactin (Dataset S4). This set of colibactin associated genes are spread across the genome, describing various biological functions including secondary metabolism, secretion system, amino acid metabolism, fimbrial usher chaperone pathways, and regulation, among others.

In total, 77 of the 88 identified colibactin-associated genes were located in 14 different genomic regions, and 11 genes were located alone throughout the genome (Dataset S4). Some of the associated genes are also conserved in part of the yersiniabactin possessing genomes (variable from 0 to 57%) revealing the importance of the associated functions during evolution for PKS-NRPS clusters in *Escherichia* (Dataset S4). The largest continuous region of 15 genes encodes for colibactin and yersiniabactin biosynthesis. These genes coded for functions including core biosynthesis, regulation and transport of colibactin and yersiniabactin. Most interestingly, one region of 14 genes coded for the assembly of a type VI Secretion System (T6SS). As described earlier in this study, we note that gene coding for assembly of another secretion system (T4SS) is present in the direct neighborhood of colibactin clusters in the genera other than *Escherichia* (Fig. 4). The specific delivery mechanism of colibactin to the host cell still remains unclear [45], with few hypotheses that include intracellular invasion of *pks+* strains [46] and outer membrane vesicles [47]. Even though T4SS and T6SS are very different, both of them share periplasm-spanning channels and secrete proteins from the cytoplasm outside the cell and across an additional host cell membrane, delivering secreted substrates directly to the cytosol of a target cell [48]. Additionally, earlier reports have shown secretion systems to be involved in secretion of the NRPS product pyoverdine in *Pseudomonas taiwanensis* [49]. It would be interesting to further investigate the role of the secretion systems in colibactin related bacterial host-communication mechanisms, especially since the cell-to-cell contact is necessary for genotoxic effects of colibactin, as discovered earlier [50,51].

Moreover, some of the other genomic regions associated with colibactin in this study also were shown to play an important role in various pathogenic *E. coli* strains. For example, a metabolic operon (region 4) with putative functions related to fructose metabolism was also shown to promote fitness under stressful conditions and invasion of host cells in extraintestinal pathogenic *E. coli* (28). In another study, additional variants of tagatose bisphosphate (TBP) aldolases, as also seen here (region 5), were associated with utilization of intestinal mucus glycan in B2 phylogroup of *E. coli* (29). One of the associate genes, *ripA_2*, coded for a transcriptional regulator of the AraC family, which was shown to be active in the iron limiting conditions [52]. The gene *ripA_2* is associatively present in all colibactin-containing strains along with the known transcriptional regulator *ybtA* of the AraC-like family. The *ybtA* gene expression is shown to be required under iron limiting conditions to initiate expression polyketides [37].

We further investigated the phylogenetic relationship to observe if the colibactin BGCs and their associated genes are evolved together. First, we calculated average nucleotide identities (ANI) of all 1,191 *Escherichia* genomes against each other using FastANI [53]. Based on the sequence similarity network of ANI and community detection algorithm [54], we selected 68 representative genomes for phylogenetic tree reconstruction using autoMLST [55]. Next, we investigated the gene presence distribution of 88 predicted colibactin associated genes across the 68 selected genomes and observed that many of these genes are mostly present in a phylogenetically closer group also harboring colibactin BGC (Fig. 5D). We also note that the genome of the strain *E. coli* RM14732, close to other colibactin-containing genomes, was missing colibactin BGC and a few of the associated gene sets, such as T6SS and d-threonate metabolism.

The identified set of associated genes suggests functions with putative roles in the overall function of a BGC. The strength of such a pangenome analysis workflow is exemplified here by the detection of putative associated genes coding for secretion systems, providing for alternative hypotheses for yet unresolved mechanisms to deliver colibactin in a contact dependent manner. Thus, the growing availability of complete genome sequences enables us to define a gene set that is associated with a particular BGC.

### 3.6 Associated genes have highly-conserved sequences in colibactin containing genomes

In the final step, the data obtained from the pangenome characterization can be used to analyze the associated genes across the pangenome of *Escherichia* with amino acid sequence level resolution. We investigated the percentage identity of amino acid sequences of associated genes from selected regions across the many genomes. For example, we discovered that the associated genes from the region encoding for the Yfc usher chaperone pathway have high conservation in the genomes possessing the PKS cluster as compared to those genomes missing the PKS cluster (Fig. S5). For such a comparison, a lower cutoff of 50% blastp percentage identity (PID) was used to define homologs for all genes in the Yfc usher chaperone pathway across 581 *Escherichia* genomes. It had been shown earlier that genes involved in usher chaperone pathways can be used for high resolution typing of uropathogenic *E. coli* [56]. Genes *YfcS, YfcQ,* and *YfcR* are especially highly conserved in the genomes possessing colibactin. In another example, the region encoding for T6SS also was found to have highly conserved sequence similarity in colibactin possessing genomes (Fig. S5). Notably, PAAR repeat protein sequences were specifically conserved in most of the colibactin possessing genomes which play an important role in diversifying the role of T6SS [57]. Thus, overview of amino acid conservation profiles of these genes demonstrate stronger association of genes with colibactin presence.

## 4 Discussion

Secondary metabolites are small molecules that play diverse roles in host-microbe and microbe-microbe interactions in enterobacteria. Earlier explorations of secondary metabolite biosynthetic potential involved studies of *Xenorhabdus sp*., *Photorhabdus sp.*, and *Serratia sp*. However, large-scale genome mining studies have not been carried out across enterobacteria. Here, we provide a comprehensive toolkit that – as one example of its application – was used to define a catalog reflecting the diversity and richness of secondary metabolite BGCs in various genera of enterobacteria. In addition to the common thiopeptides and NRPS BGC types, many other BGC types including NRPS independent siderophore synthetases, PKS-NRPS hybrids, bacteriocins, and aryl polyenes also occur frequently in enterobacterial genomes. As many secondary metabolites are involved in microbe-microbe or microbe-host interactions, the presented catalog can also be used to predict physiological properties of enterobacterial strains based on the presence of specific BGCs. For example, detection of BGCs encoding siderophores can facilitate the understanding of structural and functional variations in these iron chelating compounds. The various siderophores are important for pathogenic enterobacteria by giving them competitive advantage under metal ion limiting conditions and thus play an important role in gut.

Sequence-based similarity network analysis further detected the diverse families among each cluster type. The BGC content of enterobacteria, on average, is not as high and as diverse as more popular secondary metabolite producers such as bacilli, actinobacteria, or myxobacteria. Nonetheless, it is interesting to note that very few BGC families were assigned to known secondary metabolites, given the fact that enterobacteria have been studied so extensively for other metabolic features. This indicates a strong need to further investigate enterobacterial secondary metabolism, which can open up the possibility to discover novel secondary metabolites and their physiological roles [19]. The expression of BGCs like the colibactin BGCs is tightly regulated and depends on multiple factors, such as growth conditions, microbe–microbe or host–microbe interactions [45]. With the current state of knowledge, it is difficult to predict BGC expression under given growth conditions just based on genomic data. To gain information on BGC expression, transcriptomics, proteomics and/or metabolomics data need to be consulted [58]. Given the extensive knowledge of primary metabolism and the availability of state-of-the-art metabolic engineering tools for *E. coli*, understanding of secondary metabolism in *E. coli* and other enterobacteria can provide a guide to design host strains to efficiently express heterologous clusters from other bacteria.

The presented integrated workflow can allow to identify genes associatively present with a particular BGC using pangenome analysis tools. As an example case study, the pangenome analysis of *Escherichia* genomes revealed a set of genes that could be associated with the presence of a PKS-NRPS hybrid type cluster that codes for the genotoxin colibactin. Genes associated with the presence of colibactin BGC are spread throughout the genome and encode for diverse biological functions, such as secretion systems, amino acid metabolism, and adhesion systems among others, that are not obviously correlated with colibactin biosynthesis or activity. Amino acid sequence identity profiles further demonstrate highly conserved sequences of these associated genes in colibactin containing genomes. Very recently, the structure of the final colibactin product was revealed along with complete biosynthesis, however the export outside the bacteria still remains elusive [13,45]. Previously, a few hypotheses have been proposed [45], such as export through outer membrane vesicles [47] intracellular invasion [46]. In this study, we observed that genes coding for secretion systems are associatively present together with colibactin, presenting alternative hypotheses for possible association of secretions systems in the delivery of colibactin into host cells in a contact dependent manner. Discovery of such associations using pangenome analysis might pave the way for an understanding of the mechanisms behind clinically important colibactin and can help design strategies to target the activity of colibactin in pathogenic strains. Thus, our pangenome analysis workflow presents a platform to generate alternative novel hypotheses for BGC associations that could be the basis for further experiments confirming or disproving them.

## 5 Conclusion

In this study, we highlight a systems biology workflow that uses a growing number of genome sequences, combining genome mining and pangenome analysis tools. Here, we demonstrate that our approach can lead us to systematically analyze the secondary metabolite biosynthetic potential of a large number of genomes from a group of bacteria and discover novel associations of secondary metabolism to other functions in the genome. As sequencing technology advances and the availability of genomes continues to increase, the presented workflow can be easily extended to actinobacteria and others that produce a diverse range of secondary metabolites with antibiotic and other medicinal and industrial properties. Such an application might help us identify key enzymes or regulators that are always associatively present with the presence of a specific antibiotic encoding BGC.

## Author contributions

B.O.P and T.W. conceived the study; O.M., T.W. and B.O.P. designed the research; O.M. collected data, performed research, analyzed and interpreted the data, with assistance from C.J.L and J.M; O.M., B.O.P., T.W. drafted the article; O.M., C.J.L., J.M.M., T.W., and B.O.P. critically revised and approved the article.

## Data availability

The genome sequences used in this study are available at the PATRIC database with accessions mentioned in supplementary materials. All other data is available in the main text or supplementary materials.

## Code availability

For genome mining and generating a similarity network of biosynthetic gene clusters, we used antiSMASH v5 [3] and BiG-SCAPE [4], respectively. For pangenome analysis, the Roary pipeline [28] was used. Custom Jupyter notebooks used for data analysis and visualization of the workflow presented are uploaded at https://github.com/OmkarSaMo/Pangenome_Sec_Met.

## Declaration of competing interest

The authors declare no competing interests.

## References

[1] Cimermancic P., Medema M.H., Claesen J., Kurita K., Wieland Brown L.C., Mavrommatis K. (2014). Insights into secondary metabolism from a global analysis of prokaryotic biosynthetic gene clusters. Cell.

[2] Donia M.S., Cimermancic P., Schulze C.J., Wieland Brown L.C., Martin J., Mitreva M. (2014). A systematic analysis of biosynthetic gene clusters in the human microbiome reveals a common family of antibiotics. Cell.

[3] Blin K., Shaw S., Steinke K., Villebro R., Ziemert N., Lee S.Y. (2019). antiSMASH 5.0: updates to the secondary metabolite genome mining pipeline. Nucleic Acids Res.

[4] Navarro-Muñoz J.C., Selem-Mojica N., Mullowney M.W., Kautsar S.A., Tryon J.H., Parkinson E.I. (2020). A computational framework to explore large-scale biosynthetic diversity. Nat Chem Biol.

[5] Rouli L., Merhej V., Fournier P.E., Raoult D. (2015). The bacterial pangenome as a new tool for analysing pathogenic bacteria. New Microbes New Infect.

[6] Seif Y., Kavvas E., Lachance J.C., Yurkovich J.T., Nuccio S.P., Fang X. (2018). Genome-scale metabolic reconstructions of multiple Salmonella strains reveal serovar-specific metabolic traits. Nat Commun.

[7] Baltz R.H. (2019). Natural product drug discovery in the genomic era: realities, conjectures, misconceptions, and opportunities. J Ind Microbiol Biotechnol.

[8] Genilloud O. (2018). Mining actinomycetes for novel antibiotics in the omics era: are we ready to exploit this new paradigm?. Antibiotics.

[9] Wilson B.R., Bogdan A.R., Miyazawa M., Hashimoto K., Tsuji Y. (2016). Siderophores in iron metabolism: from mechanism to therapy potential. Trends Mol Med.

[10] Perry R.D., Fetherston J.D. (2011). Yersiniabactin iron uptake: mechanisms and role in Yersinia pestis pathogenesis. Microb Infect.

[11] Garrett W.S. (2019). The gut microbiota and colon cancer. Science.

[12] Wilson M.R., Jiang Y., Villalta P.W., Stornetta A., Boudreau P.D., Carrá A. (2019). The human gut bacterial genotoxin colibactin alkylates DNA. Science.

[13] Xue M., Kim C.S., Healy A.R., Wernke K.M., Wang Z., Frischling M.C. (2019). Structure elucidation of colibactin and its DNA cross-links. Science.

[14] Gerc A.J., Song L., Challis G.L., Stanley-Wall N.R., Coulthurst S.J. (2012). The insect pathogen Serratia marcescens Db10 uses a hybrid non-ribosomal peptide synthetase-polyketide synthase to produce the antibiotic althiomycin. PLoS One.

[15] Su C., Xiang Z., Liu Y., Zhao X., Sun Y., Li Z. (2016). Analysis of the genomic sequences and metabolites of Serratia surfactantfaciens sp. nov. YD25T that simultaneously produces prodigiosin and serrawettin W2. BMC Genom.

[16] Tobias N.J., Wolff H., Djahanschiri B., Grundmann F., Kronenwerth M., Shi Y.M. (2017). Natural product diversity associated with the nematode symbionts Photorhabdus and Xenorhabdus. Nat Microbiol.

[17] Shi Y.M., Bode H.B. (2018). Chemical language and warfare of bacterial natural products in bacteria–nematode–insect interactions. Nat Prod Rep.

[18] Tobias N.J., Shi Y.M., Bode H.B. (2018). Refining the natural product repertoire in entomopathogenic bacteria. Trends Microbiol.

[19] Maglangit F., Yu Y., Deng H. (2021). Bacterial pathogens: threat or treat (a review on bioactive natural products from bacterial pathogens). Nat Prod Rep.

[20] Imai Y., Meyer K.J., Iinishi A., Favre-Godal Q., Green R., Manuse S. (2019). A new antibiotic selectively kills Gram-negative pathogens. Nature.

[21] Wattam A.R., Abraham D., Dalay O., Disz T.L., Driscoll T., Gabbard J.L. (2014). PATRIC, the bacterial bioinformatics database and analysis resource. Nucleic Acids Res.

[22] Wu H., Wang D., Gao F. (2021). Toward a high-quality pan-genome landscape of Bacillus subtilis by removal of confounding strains. Briefings Bioinf.

[23] Wu H., Yang Z.K., Yang T., Wang D., Luo H., Gao F. (2022). An effective preprocessing method for high-quality pan-genome analysis of Bacillus subtilis and Escherichia coli. Methods Mol Biol.

[24] Stamatakis A. (2014). RAxML version 8: a tool for phylogenetic analysis and post-analysis of large phylogenies. Bioinformatics.

[25] Letunic I., Bork P. (2021). Interactive Tree of Life (iTOL) v5: an online tool for phylogenetic tree display and annotation. Nucleic Acids Res.

[26] Medema M.H., Kottmann R., Yilmaz P., Cummings M., Biggins J.B., Blin K. (2015). Minimum information about a biosynthetic gene cluster. Nat Chem Biol.

[27] Shannon P., Markiel A., Ozier O., Baliga N.S., Wang J.T., Ramage D. (2003). Cytoscape: a software environment for integrated models of biomolecular interaction networks. Genome Res.

[28] Page A.J., Cummins C.A., Hunt M., Wong V.K., Reuter S., Holden M.T.G. (2015). Roary: rapid large-scale prokaryote pan genome analysis. Bioinformatics.

[29] Antonopoulos D.A., Assaf R., Aziz R.K., Brettin T., Bun C., Conrad N. (2019). PATRIC as a unique resource for studying antimicrobial resistance. Briefings Bioinf.

[30] Kautsar S.A., Blin K., Shaw S., Navarro-Muñoz J.C., Terlouw B.R., van der Hooft J.J.J. (2020). MIBiG 2.0: a repository for biosynthetic gene clusters of known function. Nucleic Acids Res.

[31] Steinke K., Mohite O.S., Weber T., Kovács Át (2021). Phylogenetic distribution of secondary metabolites in the Bacillus subtilis species complex. mSystems.

[32] Shi Y.M., Hirschmann M., Shi Y.N., Ahmed S., Abebew D., Tobias N.J. (2022). Global analysis of biosynthetic gene clusters reveals conserved and unique natural products in entomopathogenic nematode-symbiotic bacteria. Nat. Chem..

[33] Dunbar K.L., Chekan J.R., Cox C.L., Burkhart B.J., Nair S.K., Mitchell D.A. (2014). Discovery of a new ATP-binding motif involved in peptidic azoline biosynthesis. Nat Chem Biol.

[34] Müller S.I., Valdebenito M., Hantke K. (2009). Salmochelin, the long-overlooked catecholate siderophore of Salmonella. Biometals.

[35] Lam M.M.C., Wyres K.L., Judd L.M., Wick R.R., Jenney A., Brisse S. (2018). Tracking key virulence loci encoding aerobactin and salmochelin siderophore synthesis in Klebsiella pneumoniae. Genome Med.

[36] Ozenberger B.A., Nahlik M.S., McIntosh M.A. (1987). Genetic organization of multiple fep genes encoding ferric enterobactin transport functions in Escherichia coli. J Bacteriol.

[37] Rehm N., Wallenstein A., Keizers M., Homburg S., Magistro G., Chagneau C.V. (2022). Two polyketides intertwined in complex regulation: posttranscriptional CsrA-mediated control of colibactin and yersiniabactin synthesis in Escherichia coli. mBio.

[38] Martin P., Marcq I., Magistro G., Penary M., Garcie C., Payros D. (2013). Interplay between siderophores and colibactin genotoxin biosynthetic pathways in Escherichia coli. PLoS Pathog.

[39] Wami H., Wallenstein A., Sauer D., Stoll M., von Bünau R., Oswald E. (2021). Insights into evolution and coexistence of the colibactin- and yersiniabactin secondary metabolite determinants in enterobacterial populations. Microb Genom.

[40] Garcie C., Tronnet S., Garénaux A., McCarthy A.J., Brachmann A.O., Pénary M. (2016). The bacterial stress-responsive Hsp90 chaperone (HtpG) is required for the production of the genotoxin colibactin and the siderophore yersiniabactin in Escherichia coli. J Infect Dis.

[41] Putze J., Hennequin C., Nougayrède J.P., Zhang W., Homburg S., Karch H. (2009). Genetic structure and distribution of the colibactin genomic island among members of the family Enterobacteriaceae. Infect Immun.

[42] Wallenstein A., Rehm N., Brinkmann M., Selle M., Bossuet-Greif N., Sauer D. (2020). ClbR is the key transcriptional activator of colibactin gene expression in Escherichia coli. mSphere.

[43] Bosi E., Monk J.M., Aziz R.K., Fondi M., Nizet V., Bø Palsson (2016). Comparative genome-scale modelling of Staphylococcus aureus strains identifies strain-specific metabolic capabilities linked to pathogenicity. Proc Natl Acad Sci U S A.

[44] Monk J.M., Charusanti P., Aziz R.K., Lerman J.A., Premyodhin N., Orth J.D. (2013). Genome-scale metabolic reconstructions of multiple Escherichia coli strains highlight strain-specific adaptations to nutritional environments. Proc Natl Acad Sci U S A.

[45] Dougherty M.W., Jobin C. (2021). Shining a light on colibactin biology. Toxins.

[46] Lucas C., Salesse L., Hoang M.H.T., Bonnet M., Sauvanet P., Larabi A. (2020). Autophagy of intestinal epithelial cells inhibits colorectal carcinogenesis induced by colibactin-producing Escherichia coli in apc mice. Gastroenterology.

[47] Murase K., Martin P., Porcheron G., Houle S., Helloin E., Pénary M. (2016). HlyF produced by extraintestinal pathogenic Escherichia coli is a virulence factor that regulates outer membrane vesicle biogenesis. J Infect Dis.

[48] Green E.R., Mecsas J. (2016). Bacterial secretion systems: an overview. Microbiol Spectr.

[49] Chen W.J., Kuo T.Y., Hsieh F.C., Chen P.Y., Wang C.S., Shih Y.L. (2016). Involvement of type VI secretion system in secretion of iron chelator pyoverdine in Pseudomonas taiwanensis. Sci Rep.

[50] Reuter C., Alzheimer M., Walles H., Oelschlaeger T.A. (2018). An adherent mucus layer attenuates the genotoxic effect of colibactin. Cell Microbiol.

[51] Nougayrède J.P., Homburg S., Taieb F., Boury M., Brzuszkiewicz E., Gottschalk G. (2006). Escherichia coli induces DNA double-strand breaks in eukaryotic cells. Science.

[52] Wennerhold J., Krug A., Bott M. (2005). The AraC-type regulator RipA represses aconitase and other iron proteins from Corynebacterium under iron limitation and is itself repressed by DtxR. J Biol Chem.

[53] Jain C., Rodriguez-R L.M., Phillippy A.M., Konstantinidis K.T., Aluru S. (2018). High throughput ANI analysis of 90K prokaryotic genomes reveals clear species boundaries. Nat Commun.

[54] Blondel V.D., Guillaume J.L., Lambiotte R., Lefebvre E. (2008). Fast unfolding of communities in large networks. J Stat Mech Theor Exp.

[55] Alanjary M., Steinke K., Ziemert N. (2019). AutoMLST: an automated web server for generating multi-locus species trees highlighting natural product potential. Nucleic Acids Res.

[56] Ren Y., Palusiak A., Wang W., Wang Y., Li X., Wei H. (2016). A high-resolution typing assay for uropathogenic Escherichia coli based on fimbrial diversity. Front Microbiol.

[57] Shneider M.M., Buth S.A., Ho B.T., Basler M., Mekalanos J.J., Leiman P.G. (2013). PAAR-repeat proteins sharpen and diversify the type VI secretion system spike. Nature.

[58] Palazzotto E., Weber T. (2018). Omics and multi-omics approaches to study the biosynthesis of secondary metabolites in microorganisms. Curr Opin Microbiol.

