# Supplementary material for: Pangenome analysis of Enterobacteria reveals richness of secondary metabolite gene clusters and their associated gene sets
Source: Synth Syst Biotechnol. 2022 May 6;7(3):900–10. doi: 10.1016/j.synbio.2022.04.011 (PMC9125672; doi:10.1016/j.synbio.2022.04.011)
Supplement: Multimedia component 5 [file mmc5.pdf]

Supplemental Material for

Pangenome Analysis of Enterobacteria Reveals Richness of Secondary Metabolite Gene Clusters and their Associated Gene Sets

Omkar S. Mohite[1], Colton J. Lloyd[2], Jonathan M. Monk[2], Tilmann Weber[1]*, Bernhard O. Palsson[1,2]*.

[1]The Novo Nordisk Foundation Center for Biosustainability, Technical University of Denmark, Kongens Lyngby, Denmark

[2]Department of Bioengineering, University of California, San Diego, La Jolla, USA.

***Correspondence:** tiwe@biosustain.dtu.dk

This PDF file includes:

Figures S1 to S5

Legends for Datasets S1 to S4

Other supplemental materials for this manuscript include the following:

Datasets S1 to S4

Figure S1

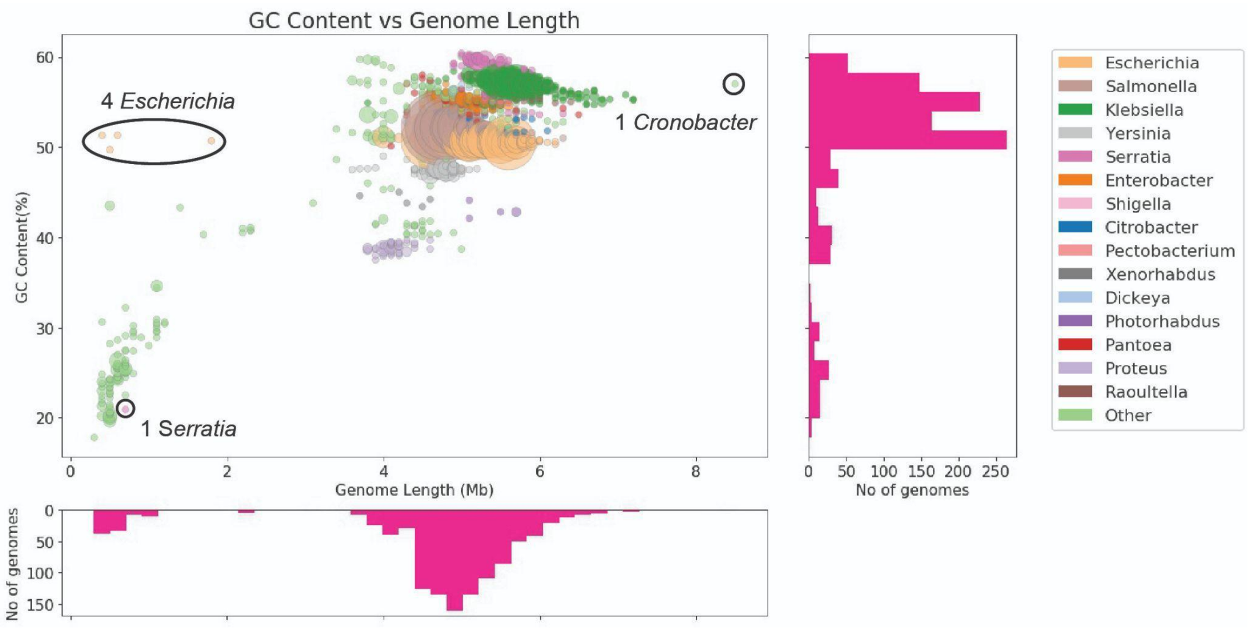

Figure S1. Overview of genome characteristics of the enterobacteria genomes and curation

Scatter plot showing the distribution of genome length and GC content of 3895. The size of the circles represents the number of genomes, colors denote major genera. Associated histograms represent the distribution of BGCs in genomes (right) and lengths of genomes (bottom). Highlighted 4 genomes of *Escherichia,* one genome of each *Serratia* and *Cronobacter* are removed from the dataset due to their atypical genome size. (Look at Dataset S1 for accession IDs of input genomes and filtered genomes)

Figure S2

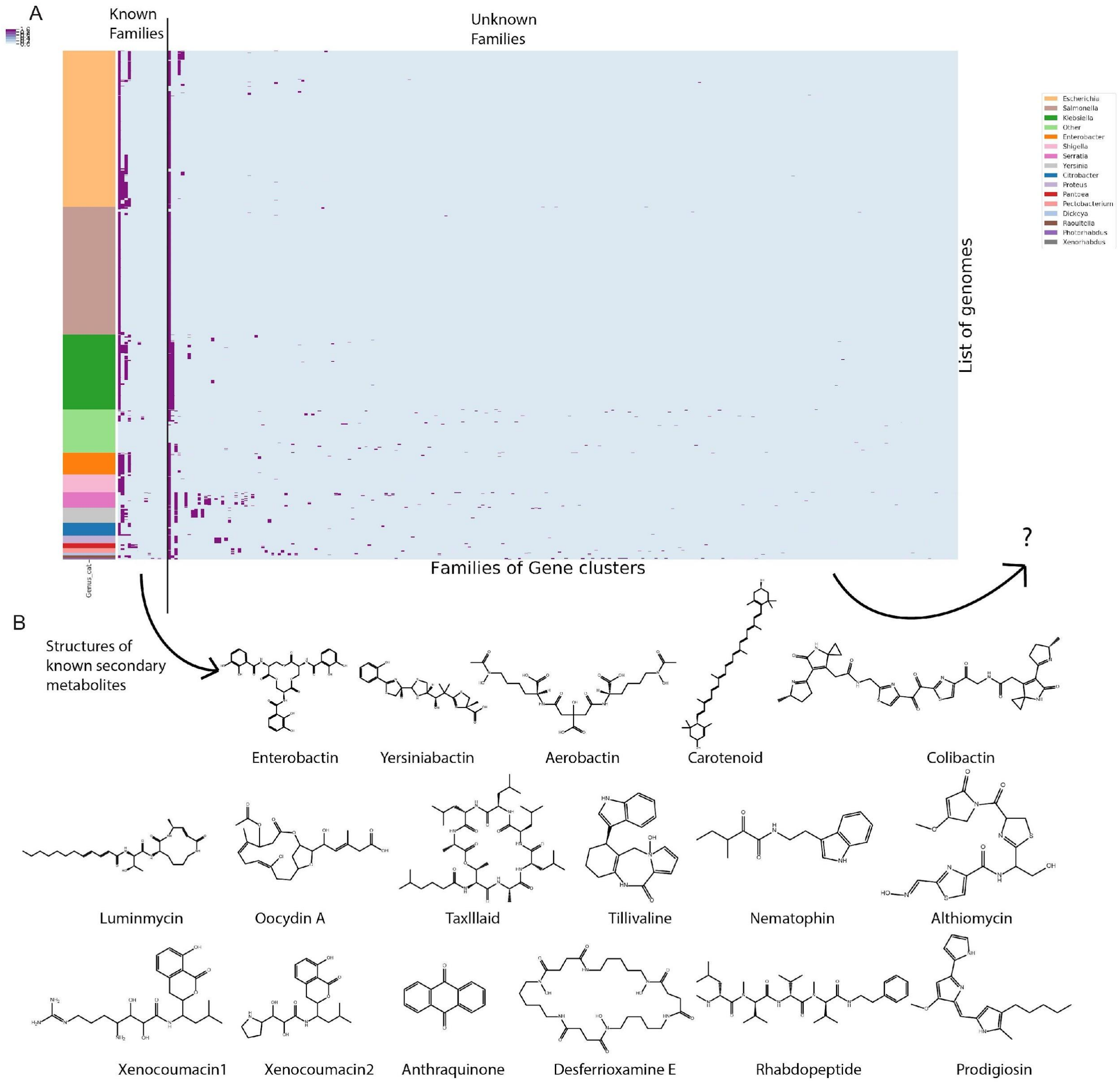

Figure S2. Distribution of BGC families across genomes

A. Distribution of families of BGCs across genomes from different genera. The rows of heatmap represent 3889 genomes ordered according to the genus they belong to. The columns represent the presence of a BGC from one of the 252 distinct families that have at least two BGCs. The columns are split into families that have been associated with known BGCs from MIBIG and others, which are new families, detected in this study. B. The structures of some secondary metabolites encoded by known BGCs.

Figure S3

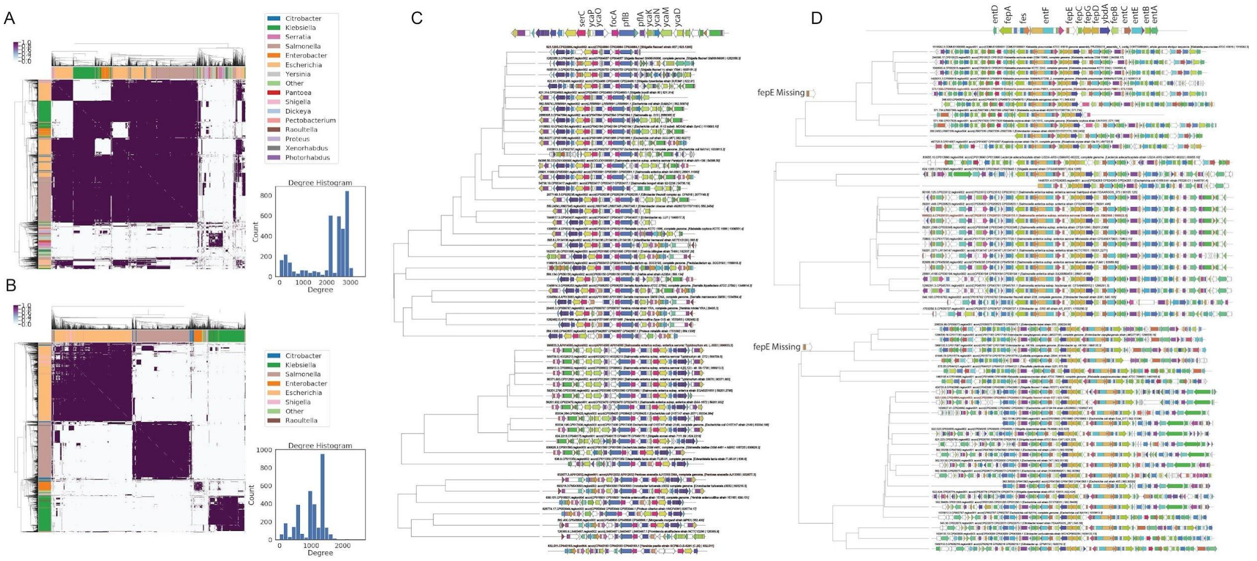

Figure S3. BGC variation across genera within families

The adjacency matrix of the similarity network of family 2 (panel A) and family 3 (panel B). The row and column colors of heatmap represent the different genera of the genome with this BGC. Degree histograms are represented in bottom right box to show highly connected network. Note that a cutoff of 0.3 was used for generating similarity networks this the nodes with distance above 0.3 are considered disjoint. Alignment of selected BGCs from family 2 (panel C) and family 3 (panel D) showing minor variations in genetic structure of these BGCs, which are mostly highly conserved. Core genes of biosynthesis such as *ycaO* of family 2 and enterobactin biosynthetic genes of family 3 are present across all BGCs within each family. However, gene *fepE* which is part of ferric enterobactin transport systems, was missing in multiple BGCs within family 3.

Figure S4

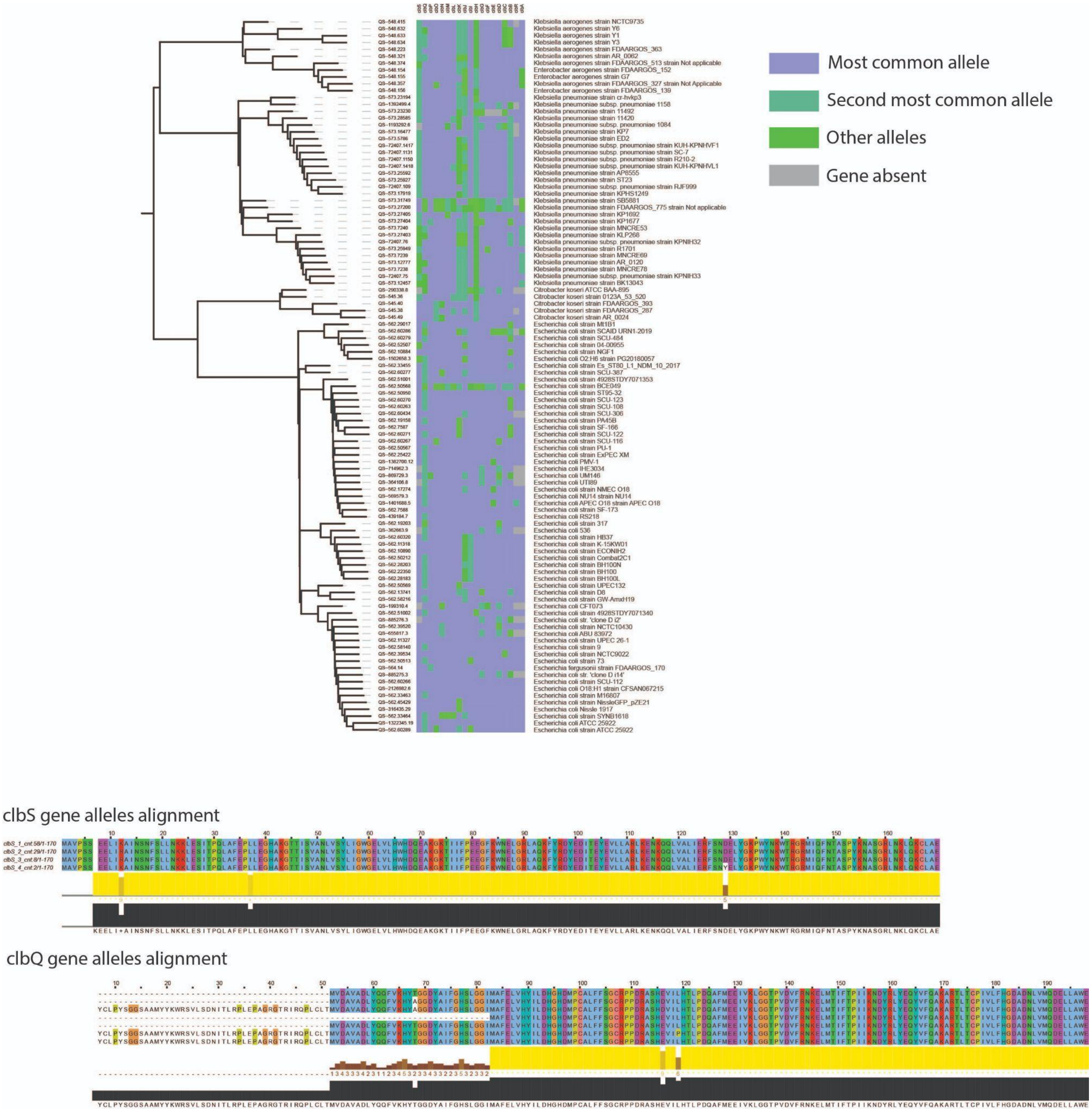

Figure S4. Genetic variation within colibactin biosynthetic genes across family of BGCs

Heatmap displaying presence of various alleles across 104 colibactin containing geomes sorted in the phylogenetic order. Most common alleles are displayed by colors in the heatmap. Amino acid sequence variations for different alleles of genes *clbS* and *clbQ*

Figure S5

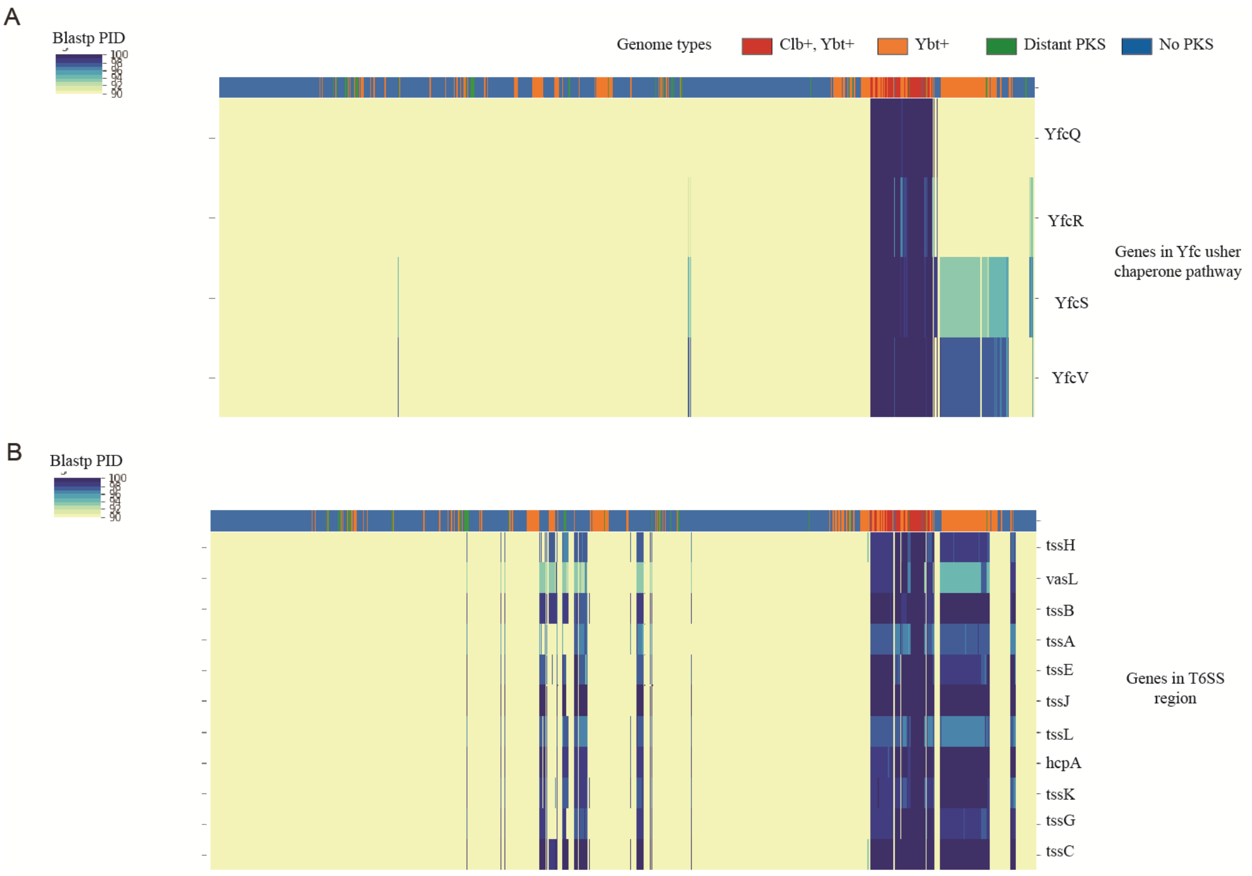

Figure S5. Blast PID distribution of associated genes across genomes

Percentage blast similarity (for PID > 95%) distribution for homologs of associated genes from regions encoding for a) Yfc usher chaperone pathway and b) type VI secretion system (Dataset S4). Columns represent all 1,192 genomes of *Escherichia* in phylogenetic order.

Dataset S1. Input dataset of enterobacterial genomes from PATRIC database

List of 4035 genomes that were classified as complete at PATRIC database (sheet: PATRIC_complete). The list of 3987 genomes downloaded with all the associated metadata that is present in the PATRIC database. Genomes with complete status and single coting chromosome assembly were chosen (sheet: downloaded_genomes). List of genomes that are removed from the analysis after manual curation (Methods) (sheet: manual_curation). Final list of genomes in the curated set used for analysis (sheet: input_genomes).

Dataset S2. Secondary metabolite BGCs detected across 3,889 genomes using antiSMASH

List of 13,266 BGCs detected using antiSMASH with details of BGC type, position, genome data and contig data where BGCs are found (sheet: detected_bgcs). List of 99 BGCs on the contig edge that are removed from the analysis (Methods) (sheet: removed_bgcs_on_contig_edge). Number of BGCs of different types as defined by antiSMASH rule-based detection logic (sheet: bgc_count_type). Please note that, here 'other' represents a BGC type (not to be confused with 'Other' types defined for Figure 1) that is not assigned to well-known BGCs as per antiSMASH BGC detection logic. List of 50 manually selected enterobacterial genomes from various genera with different distributions of BGC types used as in-group for construction of phylogenetic tree using maximum likelihood algorithm (sheet: phylo_tree_accn). List of five genomes from neighboring clades of Gammaproteobacteria used as out-group during phylogenetic tree construction (Dataset S2) (sheet: phylo_tree_accn).

Dataset S3. Sequence-based similarity network of BGCs

List of 3110 BGC nodes from families 8 to 599 leading to the similarity network displayed in Figure 2 (sheet: node_table_small_families). The various sequence similarity distances generated using BiG-SCAPE, the raw distance metric was used to define edges of the network (sheet: edge_table_small_famillies). List of BGCs in top 7 largest families (sheet: large_families_cluster_list). List of 105 colibactin BGCs detected across genera and

families detected using BiG-SCAPE (sheet: colibactin_bgcs). The various sequence similarity distances generated using BiG-SCAPE. The raw distance metric was used to define edges of the network (sheet: edge_table_colibactin_bgcs). Four of the BGCs from different genera and different families detected here are visualized in Figure 3. Presence of core biosynthetic gene of colibactin sheet: colibactin_biosynthetic_ortho). Gene allele variation analysis for colibactin biosynthetic genes (sheet: colibactin_allele_count).

Dataset S4. Pangenome analysis of *Escherichia* genomes to identify colibactin BGC associated genes

List of 60 *Escherichia* genomes with colibactin BGC that are selected for pangenome analysis (sheet: colibactin_containing_ecoli). Total list of 17,728 genes detected in the pangenome of 60 *Escherichia* genomes reconstructed using Roary software. Gene presence-absence tables across 60 genomes (sheet: gene_presence_absence/boolean). Percent identity score of bidirectional best blast hits of 2530 genes from the core genome of colibactin containing *Escherichia* against 1,191 genomes of *Escherichia* (sheet: clb_vs_all_ecoli_bbh). List of 88 genes that are associatively present in Colibactin containing *Escherichia* genomes (sheet: associated_genes). Regions are defined if two or more neighboring genes are present in associated gene sets. Additionally, corresponding gene accession IDs for well-characterized strain *E. coli* CFT073 are listed.
